# Decreased quality of life and societal impact of cryopyrin-associated periodic syndrome treated with canakinumab: a questionnaire based cohort study

**DOI:** 10.1186/s13023-018-0799-1

**Published:** 2018-04-20

**Authors:** Catharina M. Mulders-Manders, Tim A. Kanters, Paul L. A. van Daele, Esther Hoppenreijs, G. Elizabeth Legger, Jan A. M. van Laar, Anna Simon, Leona Hakkaart-van Roijen

**Affiliations:** 1 Department of Internal Medicine, Radboud university medical center, 463, PO box 9101, 6500 HB Nijmegen, the Netherlands; 2 Expertise Center for Immunodeficiency and Autoinflammation, Radboud university medical center, 463, PO box 9101, 6500 HB Nijmegen, the Netherlands; 3 Erasmus University Rotterdam, Institute for Medical Technology Assessment, Bayle Building - Campus Woudestein, PO box 1738, 3000 DR Rotterdam, The Netherlands; 4 Department of Internal Medicine and Immunology, Erasmus MC, Front Service Immunology, room: Nb-1218, PO box 2040, 3000 CA Rotterdam, the Netherlands; 5 Department of Paediatric Rheumatology, Radboud university medical center, Amalia Children’s Hospital, 804, PO box 9101, 6500 HB Nijmegen, the Netherlands; 6 Department of Pediatrics, University Medical Center Groningen, PO box 30.001, 9700 RB Groningen, the Netherlands

**Keywords:** Cryopyrin-associated periodic syndrome, Muckle Wells syndrome, Neonatal onset multisystem inflammatory disease, Chronic infantile neurologic cutaneous and arthritis syndrome, Canakinumab, Anakinra, Quality of life, Societal impact

## Abstract

**Background:**

Cryopyrin-associated periodic syndrome (CAPS) is a rare disease. Knowledge on the quality of life (QoL) and the disease’s societal impact is limited. Canakinumab is used in increasing frequency for the treatment of CAPS.

**Methods:**

Observational study in Dutch CAPS patients. Patients completed questionnaires regarding treatment with canakinumab at baseline and retrospectively. Quality of life was assessed using the EQ-5D-5L in adults and CHQ-PF50 in children. Impact on work and school was assessed. Caregivers' quality of life was assessed using the CarerQol.

**Results:**

Mean quality of life scores during treatment with canakinumab were 0.769 (EQ-5D-5L), 51.1 (CHQ-P) and 57–1 (CHQ-M). Most patients experienced problems on the pain/discomfort dimension. Higher disease activity and the presence of complications negatively influenced QoL. Half of the patients with a paid job reported absenteeism from work due to CAPS, for an average of 8.7 days in a 4-week period. All schoolgoing patients (*N* = 5) reported absence from school due to CAPS, for an average of 2.9 days. Caregivers reported gaining a lot fulfillment from providing care for their family members.

**Conclusion:**

QoL during treatment is lower than in the general Dutch population. CAPS leads to productivity loss and absenteeism from school, and impacts the quality of life in informal caregivers.

**Electronic supplementary material:**

The online version of this article (10.1186/s13023-018-0799-1) contains supplementary material, which is available to authorized users.

## Background

The cryopyrin-associated periodic syndrome (CAPS) is an ultra-orphan disease [1, 2]. It is one of the classic monogenic autoinflammatory diseases and forms a spectrum of disease with various phenotypes, characterized by recurrent or continuous inflammation associated with fever, arthralgia or arthritis, skin rash, neurologic abnormalities, and many other symptoms [3]. CAPS is caused by autosomal dominant mutations in the gene encoding the *nucleotide-binding oligomerization domain, leucin-rich repeat and pyrin domain containing 3 protein* (NLRP3). This protein forms the cornerstone of the NLRP3-inflammasome, a protein complex involved in the innate immune system. Activation of the NLRP3-inflammasome leads to overproduction and activation of the pro-inflammatory cytokine interleukin-1 beta (IL-1β) [3]. Because of this, IL-1 inhibition with anakinra or canakinumab is the most effective treatment for CAPS [4].

Efficacy and side effects for canakinumab and anakinra are comparable [5]. Treatment with canakinumab is over ten times as expensive as treatment with anakinra [6]. The primary aim of the current study is to quantify quality of life in patients with CAPS. Secondary aim was assessment of the broader societal impact of CAPS on patient’s work and school.

## Methods

### Patient population

All patients with a clinical diagnosis of CAPS in the Netherlands were eligible to participate in the study. In 2011, canakinumab was given an orphan designation for the treatment of CAPS in the Netherlands. Consequently, canakinumab could only be prescribed in two tertiary referral centers: the Radboud university medical center (Radboudumc, Nijmegen, the Netherlands) and the Erasmus medical center (Erasmus MC, Rotterdam, the Netherlands). As a result of this rule, all CAPS patients currently using or ever having used canakinumab were regularly evaluated in these hospitals. Since 2016, canakinumab can also be prescribed in other centers. Patients that had not been treated in the Radboudumc or Erasmus MC were identified through personal communication with consultants in other Dutch tertiary referral centers.

This study was assessed by the local medical ethics committees of the Radboudumc (registration number 2015–1643), Erasmus MC (registration number 2015–501), and university medical center Groningen (registration number 2015/166) and according to Dutch law was exempt from approval by all three, because of its retrospective and observational design and the anonymous storage of patient data. All patients or their parents gave written informed consent to participate in this study.

### Instruments

#### Quality of life in CAPS patients

Quality of life in patients above the age of 12 was assessed using the Euroqol EQ-5D-5L instrument [7]. Quality of life was expressed in utilities. A utility is an index value of health, commonly standardized between 1 (perfect health) and 0 (dead). Utilities are not disease-specific, allowing comparison between different diseases and health care interventions. Dutch tariffs were used to derive utilities from the EQ-5D-5L. EQ-5D-5L utilities range from − 0.466 to 1.00 [8].

For children < 18 years the Child Health Questionnaire, Parent Form 50 (CHQ-PF50) [9] was completed by the patient’s parents. Although this questionnaire is not validated for children less than 5 years of age, it was used anyway as no alternative validated questionnaires for quality of life are available for this age group. Patients between the ages of 12 and 18 years or their parents completed both the CHQ-PF50 and EQ-5D-5L questionnaires. The CHQ-PF50 can be transformed into two summary measures: physical and psychosocial functioning, for which a score of 50 represents the average norm-based score in the US population [10].

#### Quality of life in caregivers

Quality of life of informal caregivers was assessed using the CarerQol instrument [11]. An overall quality of life score can be derived using a Dutch tariff (i.e. a set of regression coefficients derived from a valuation study), which transforms quality of life to a scale from zero (worst situation) to 100 (best situation) [12].

#### Impact on school and work

For patients that went to school, impact of CAPS on school was assessed measuring the number of days absent from school. For patients with a job, CAPS’ impact on work was assessed using the iMTA productivity cost questionnaire (iPCQ) [13].

#### CAPS symptoms

Epidemiologic data and data on CAPS phenotype were collected from the medical record by one of the investigators. CAPS disease activity was prospectively measured upon entering the study using the Auto-Inflammatory Disease Activity Index (AIDAI) questionnaire [14]. The corresponding total AIDAI score ranges from zero to 13 points per day, with higher scores indicating more severe disease activity. This questionnaire was filled out during two consecutive months. As an alternative, a modified version of AIDAI via an online questionnaire was made available, in which all criteria from the conventional AIDAI were scored on a monthly basis during 12 consecutive months. Patients were free to choose between using the conventional or online AIDAI. Patients using the online version received a monthly email reminder containing a link to the questionnaire.

### Data collection

Data were collected between April 2015 and April 2017. For each individual patient, total study duration was 1 year, during which patients were asked to complete all questionnaires on quality of life and production loss described above two times: at baseline (*T* = 0), and a second time, looking back on the time before they used canakinumab. All questionnaires, with exception of the online AIDAI, were sent by mail and included a detailed instruction letter and pre-paid return envelope. If patients did not return their questionnaires within 2 months, they received a reminder via telephone, post, or email.

### Data analysis

Analyses were performed in Stata, version 14.1 (StataCorp, 2015). Due to the limited number of patients in the sample, no statistical tests were performed.

## Results

### CAPS population

A total of 40 CAPS patients were identified during the study, of which 31 were adults (77.5%), five adolescents aged 12 to 18 years (12.5%), and four children younger than 12 years (10.0%). Twenty patients (50%) were male. The total prevalence of CAPS in The Netherlands is 1 in 435.000 inhabitants. Twenty-four of the 40 CAPS patients (60.0%) were included in the study. The main reason for exclusion was the absence of informed consent (12 patients, 30.0%). One patient was excluded because of a language barrier, and another patient was lost to follow up. Two additional patients were not included as they came to the attention of the investigators after the inclusion period of the study had been closed. Median age at the start of the study was 28.5 years (range 5–82 years). Over 50% of patients had CAPS-related complications (Table 1).

**Table 1:** Patient characteristics and prevalence of CAPS symptoms

	Included in study
Number of patients	24
Male gender	9 (37.5%)
Age group	
Child (0–12 years)	4 (16.7%)
Adolescent (12–18 years)	3 (12.5%)
Adult (18 years or older)	17 (70.8%)
Age (years, median (range))	
Onset	5 (0–54)
Diagnosis	20 (1–76)
Start anti-IL-1	19 (1–76)
Start study	28 (5–82)
T0 treatment	
No	2 (8.3%)
Anakinra	3 (12.5%)
Canakinumab	19 (79.2%)
Duration of treatment at T0 (months, median (range))	
Anakinra	46 (42–48)
Canakinumab	49 (1–76)
Dose (mg, median (range)))	
Anakinra	100
Canakinumab	150 (45–300)
Dose interval (median, (range))	
Anakinra (days)	1
Canakinumab (weeks)	8 (4–10)
Pattern	
Continuous	5 (20.8%)
Episodic	10 (41.7%)
Continuous with flares	9 (37.5%)
NLRP3 mutation	
V198 M	5 (20.8%)
T348 M	3 (12.5%)
Y859H	3 (12.5%)
W414 L	3 (12.5%)
A439V	2 (8.3%)
R488K	1 (4.2%)
No mutation	7 (29.2%)
Symptoms	
Fever	13 (54.2%)
Skin rash	20 (83.3%)
Musculoskeletal complaints	21 (87.5%)
Ocular symptoms	13 (54.2%)
Neurologic symptoms	16 (66.7%)
Gastrointestinal symptoms	8 (33.3%)
Lymphoreticular symptoms	4 (16.7%)
Cardiopulmonal symptoms	3 (12.5%)
Other symptoms	8 (33.3%)
Complications	13 (54.2%)

Out of the 24 patients included in the study, 17 completed the quality of life questionnaires and could be included in the analysis: three children, two adolescents, and 12 adults. There were no significant differences between the entire Dutch CAPS population and the study population (Additional file 1).

### Quality of life in CAPS patients during treatment with canakinumab

At the start of the study, the average EQ-5D-5L quality of life for adults and adolescents receiving canakinumab (*N* = 14) was 0.769 (SD = 0.178).(Table 2) One female patients had a utility value of 1.000, indicating perfect health despite her disease. The minimum EQ-5D score was 0.296, observed in one male patient. None of the patients reported to have extreme problems on any of the dimensions of the EQ-5D. For both self-care and anxiety/depression, 86% of patients reported no problems.

**Table 2:** Quality of life in 24 CAPS patients at baseline

Patient	Age (years)	Gender	Disease duration to start T0 drug (years)	Drug^a^	Treatment duration (months)	EQ-5D	CHQ-physical^b^	CHQ-psycho social^b^	AIDAI
1001	62.6	M	26.3	Cana 150q6w	51	0.296			9.7
1002	39.0	M	UK	Cana 150q6w	51	0.786			
1003	59.2	F	38.8	Cana 150q8w	16	0.848			4.2
1004	59.2	F	0.33	Cana 150 q8w	57				
1005	34.7	F		No					
1006	28.9	F	UK	Cana 150q8w	1	0.680			
1007	61.2	F	UK	Cana 150q8w	10	0.813			
1008	65.0	M	UK	Cana 150q8w	76	0.533			0.0
1009	69.1	M	UK	Cana 150q8w	55	0.852			
2001	24.6	M	20.25	Cana 150q8w	51	0.887			0.1
2002	65.8	M	41.5	Cana 150q8w	51	0.695			
2003	82.0	M	48.4	Ana 100q1d	42				
2004	52.0	F	39.3	Cana 300q4w	56	0.848			2.8
2005	54.6	F	UK	Cana 150q10w	33	0.752			0.4
2006	5.6	M	4.7	Cana 100q8w	10				
2007	15.5	F	11.4	Cana 150q10w	48	1.000	58.7	54.3	
2008	5.3	F	2.1	Cana 45q8w	29		46.9	57.7	0.1
2009	23.0	F		No					
2010	8.2	F	4.1	Cana 90q8w	48		49.1	59.3	0.0
2011	8.4	F	3.0	Cana 50q8w	20		52.7	61.7	0.0
2012	16.0	M	UK	Ana 100q1d	48				
2013	16.2	F	4.7	Cana 150q10w	49	0.887	47.9	57.1	
2014	24.4	F	19.1	Cana 150q8w	57	0.887			3.4
2015	23.1	F	19.2	Ana 100q1d	46				
Median					48				
Mean						0.769	51.1	57.1	2.1

*Ana* anakinra, *cana* canakinumab, *d* day, *q* each, *w* week

^a^Drug doses are in mg per time interval, e.g. 150q8w = 150 mg every 8 weeks

^b^CHQ-PF was only performed in patients < 18 years of age

**Fig. 1:**
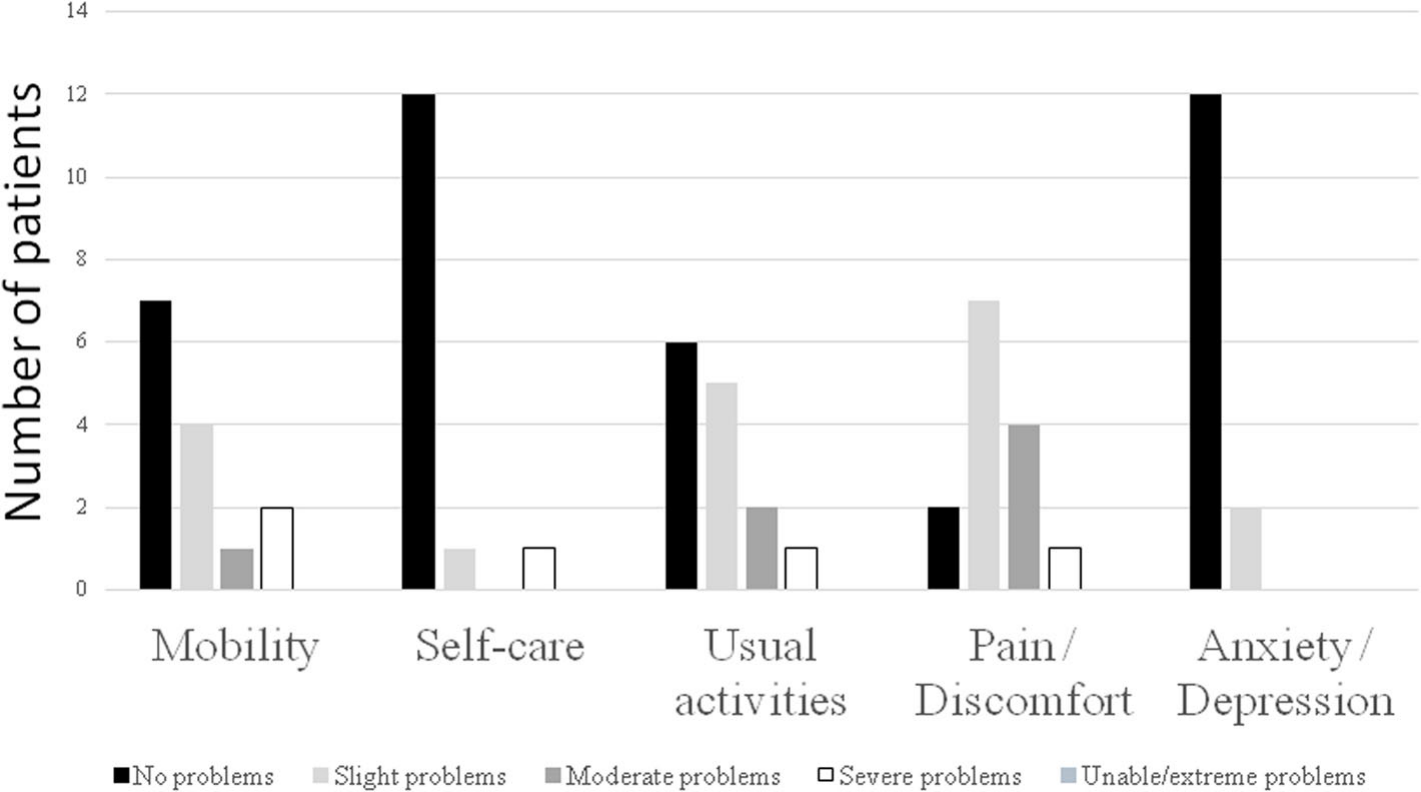
Performance of CAPS patients on the dimensions of the EQ-5D-5 L

Patients reported most problems on the pain/discomfort dimension, for which 86% of patients reported to have a problem. (Fig. 1).

For five paediatric patients data on quality of life during treatment with canakinumab were available at *T* = 0 (Table 2). The average score on CHQ-physical (51.1) and CHQ-psychosocial (57.1) were close to the standardized score of 50 in the general US population. Variation in CHQ scores between patients was limited (range CHQ-physical 46.9–58.7, range CHQ-psychosocial: 54.3–61.7).

### Quality of life during treatment with anakinra

Eight patients completed the questionnaire recalling their experiences during treatment with anakinra. In all these patients, anakinra had been stopped when canakinumab was started, and the median time between the stop of anakinra and the questionnaire was 49.5 months (range 1–76 months). At *T* = 0, three patients reported higher quality of life during canakinumab treatment, while two patients reported higher quality of life during treatment with anakinra. Three patients reported no differences in quality of life during both treatments. On average, quality of life was higher during treatment with canakinumab than during treatment with anakinra, with an average difference of 0.110 between the two treatments. Variation in quality of life differences was substantial (SD = 0.269).

### Quality of life and disease activity

Quality of life values were negatively correlated to AIDAI scores (*r* = − 0.572, *n* = 8); patients with higher disease activity had lower quality of life scores. (Table 2).

Patients with CAPS-related complications had lower quality of life than patients without complications (0.720 ± 0.184 versus 0.890 ± 0.078 at *T* = 0).

### Impact of CAPS on school and work

A total of five patients were attending school: three went to primary school, one to secondary school, and one to higher education. All of these patients had called in sick at least 1 day (average 2.9 days) in a 4-week period.

Six of the adult patients had a paid job or were self-employed, two patients were retired and one patients had been declared unfit for work. Three of the six patients with a paid job reported they had been absent from work in a 4 week period, with an average of 8.7 days. Three patients were less productive at work and three patients were unable to perform all their unpaid work activities (such as household or volunteer work).

### Caregivers’ quality of life

Six caregivers were included in the study. Five were the patient’s partner and one was the patient’s mother. On average, these caregivers provided 9 hours of caregiving per week, particularly related to household activities and social interaction. Of all caregivers, 83% derived a lot of fulfillment from providing informal care. None of the caregivers reported severe problems on any of the domains of the CarerQol. Three (50.0%) reported mental health problems related to their caregiving. The average CarerQol score was 87.8 (SD = 12.7) and the minimum score was 64.4. One caregiver reported the maximum quality of life score of 100. CarerQol score was strongly and positively correlated with the patient’s quality of life (*r* = 0.666).

## Discussion

This questionnaire-based study assessed health related quality of life of patients with CAPS, most of whom had been treated with canakinumab. Median treatment duration was four years. The average quality of life was 0.769, which is lower than the average utility value for the general Dutch population (corrected for gender: 0.87) [8]. CAPS-related complications and higher disease activity negatively influence quality of life. Absenteeism from school or work is frequent in patients with CAPS.

The current study is the first to report utility values for patients with CAPS, and therefore direct comparison to other studies is impossible. In children with CAPS, the Physical CHQ scores found in our study were similar to those from a previous study in CAPS patients treated with canakinumab [15]. The average psychosocial CHQ score was higher in our study, but it should be noted that the CHQ was only available for five patients. The impact of CAPS on quality of life lies between that of patients with rheumatoid arthritis (average EQ-5D-5 L utility value 0.43–0.75) and type 1 diabetes mellitus (average EQ-5D-5 L utility value 0.88) in the Netherlands [16, 17].

A phase III study showed that quality of life in CAPS patients approached values of the general population after 8 days, 8 and 48 weeks of treatment with canakinumab [18]. Another study assessed the quality of life in predominantly paediatric CAPS patients, and showed that patients who received canakinumab had quality of life scores similar to healthy controls after 12 weeks of treatment [15]. In our study we found that quality of life was less in CAPS patients than in the general population. The longer treatment duration in our study compared to the other studies, the fact that the other two were clinical research studies, in which more intensive disease activity monitoring may lead to improvements in disease control and consequently quality of life, or the use of different questionnaires may explain the different results.

Eight patients completed a retrospective questionnaire recalling their quality of life during treatment with anakinra, with a median interval between the stop of anakinra and the questionnaire of 49.5 months. Their average EQ-5D value during anakinra treatment was 0.756, which is similar to the overall population. When compared to treatment with anakinra, quality of life on average was higher during treatment with canakinumab. The current results comparing quality of life between treatment with anakinra and canakinumab may be influenced by the retrospective nature of the questionnaire used, which may induce recall bias, and the small number of patients reporting their experiences with anakinra. However, as almost all patients in the Netherlands are currently on canakinumab, it was not possible to prospectively compare quality of life between treatment with anakinra and canakinumab.

There are no other studies directly comparing QoL in CAPS patients treated with anakinra or canakinumab. Kuemmerle-Deschner et al. compared the effectiveness of treatment with anakinra and canakinumab in patients with CAPS [5]. Of the 12 patients treated with anakinra, 75% had clinical and biochemical remission after a median follow up of 50 months, while 93% of the 14 CAPS patients treated with canakinumab showed remission after a median follow up of 52 months. As this study shows that canakinumab is possibly more effective than anakinra in patients with CAPS, this could explain the higher quality of life during canakinumab treatment. Quality of life was not assessed in this study.

In a quality of life study including 13 paediatric CAPS patients treated with anakinra, both the physical summary score and the psychosocial summary score for the CHQ-PF50 increased significantly during a median follow up of 37.5 months and showed normalization of most physical activities. On the physiological domains, patients scored less beneficial on parental impact time and family activities [19]. This could be explained by the fact that treatment with anakinra requires daily subcutaneous injections, which, in case of children, must be administered by the caregiver, where canakinumab is given once every 4 to 8 weeks or even less frequently.

Although treatment with IL-1 blocking agents may be highly effective [5], patients with CAPS are often absent from work or school. CAPS negatively influences caregivers’ quality of life, and their quality of life depends on CAPS disease activity.

A recent French study also assessed quality of life in CAPS patients who had received at least one dose of canakinumab, using a descriptive interview, and found that the start of canakinumab was associated with an increase in different social activities. Family relations, friendships, and love lives were considered more fulfilling in approximately 40% of patients [20]. This study also reported that initiation of canakinumab treatment was associated with less absenteeism from school, less learning difficulties, or grade repetitions. Adults were less absent from work than before canakinumab treatment. Although this study shows that the start of canakinumab is associated with increases in different aspects of social lives, it also shows that, even when treated with canakinumab, CAPS keeps having an influence on quality of life and the daily life activities of patients. Results from this study are not directly comparable to our results, as different questionnaires were used. Also, we studied patients currently using canakinumab, where the French study included patients that had ever received one or more doses of canakinumab.

In our study, the CarerQol score for informal caregivers of CAPS patients was 87.8, which is higher than the average score of 79.1 for caregivers in the general Dutch population [21]. Still, half of the caregivers in our study reported mental health problems related to providing care. Only a small number of informal caregivers were included in the study. This is due to the fact that some of the patients do not receive informal care as they are fully independent in their daily activities, that they do not recognize informal care as such, or that the caregiver does not recognize their help for the patient as informal care. Koné-Paut et al. found that informal caregivers of CAPS patients on average spent half an hour per week on informal care, where we found that caregivers provided 9 hours of care per week. This difference could be explained by the use of different questionnaires with different definitions of informal care, or the small number of caregivers in our cohort. We found that caregiving led to fulfillment for many of the caregivers. In the French study, the start of canakinumab was associated with an increase in free time for the caregiver, and a decrease in absenteeism from work for the caregiver [20].

Our study has several limitations. Studies in orphan diseases are necessarily done in small study populations. In this study 60.0% of the total Dutch CAPS population of 40 patients was included. International data collection could increase sample size and may lead to more accurate estimates of quality of life. However, it may be hard to compare such data as quality of life or effects on life, school or work between international cohorts because of cultural differences. The observational design of this study entails that selection bias cannot be ruled out. It could be possible that patients participating in the study may differ from patients that declined to participate. There were no differences in gender or age between participants and non-participants, but the treating physicians of the patients confirm that some of the patients that declined participation had a more severe CAPS phenotype. Measurement of quality of life can only be done using questionnaires, which will not encompass all aspects influencing quality of life. We tried to overcome this limitation as much as possible by using validated quality of life questionnaires. Quality of life may be influenced by either the disease, its treatment, or a combination of both, and which factor influences QoL the most cannot be derived from our study. For the retrospective part of this study no validated questionnaires were available, possibly influencing the results. Also, patients had been treated with canakinumab for a median of approximately 4 years. The long interval between the current and the previous treatment could lead to recall bias. Because of the observational design of the study and the fact that only five of the 24 patients in our cohort did not use canakinumab, no prospective control arm for comparison of quality of life between different treatments was available.

With the introduction of a growing number of (expensive) orphan drugs, research in rare diseases is becoming increasingly relevant. The results from this study quantify the burden of disease for patients with CAPS during treatment with canakinumab and the implications for their direct environment. We show that quality of life measured with the EQ-5D-5L is an accurate way to assess disease severity in CAPS, as quality of life correlated with disease activity measured with AIDAI. The utility values derived in this study are important input values for future cost-effectiveness studies. For cost-effectiveness studies, it is necessary to divide patients into multiple distinct health states for modeling. The small sample size in orphan diseases in general and CAPS in specific may lead to implausible quality of health values. Future results could investigate cost-effectiveness and could include research on cost of medication. The current study shows that including a societal perspective is important, as CAPS patients report that their disease has significant impact on work, ability, school attendance, and the use of informal care. Costs outside the health care factor, such as the costs associated with informal care and loss of productivity, should be taken into account.

## Conclusion

Quality of life in patients with CAPS during treatment with anti-IL-1 therapy is decreased compared to quality of life in the general population. CAPS is associated with production losses in adult patients and absenteeism from school in children. Informal caregivers of patients with CAPS have higher care-related quality of life than other Dutch informal caregivers. Because of this, adequate disease control and attention to the societal impact of the disease and its treatment are pivotal in the care for patients with CAPS.

## Additional file

Additional file 1 Detailed overview of CAPS symptoms and complications in 24 Dutch CAPS patients. (DOCX 14 kb)

## Abbreviations

AA: Amyloid A
AIDAI: Autoinflammatory disease activity index
CAPS: Cryopyrin-associated periodic syndrome
CarerQol: Caregiver quality of life questionnaire
CHQ-PF: Child health questionnaire, parent form 50
CINCA: Chronic infantile neurologic, cutaneous and arthritis syndrome
EQ-5D-5L: Euroqol 5 dimensions, 5 level questionnaire
Erasmus MC: Erasmus medical center
FCAS: Familial cold autoinflammatory syndrome
IL-1 β: Interleukin-1 beta
iMTA: Institute for Medical Technology Assessment
MWS: Muckle Wells syndrome
NLRP3: Nucleotide-binding oligomerization domain, leucin-rich repeat and pyrin domain containing 3 protein
NOMID: Neonatal onset multisystem inflammatory disease
PCQ: Productivity cost questionnaire
QoL: Quality of life
Radboudumc: Radboud university medical center
