# Supplementary material for: Decreased quality of life and societal impact of cryopyrin-associated periodic syndrome treated with canakinumab: a questionnaire based cohort study
Source: Orphanet J Rare Dis. 2018 Apr 20;13:59. doi: 10.1186/s13023-018-0799-1 (PMC5910566; doi:10.1186/s13023-018-0799-1)
Supplement: Supplementary file 1 — Detailed overview of CAPS symptoms and complications in 24 Dutch CAPS patients. (DOCX 14 kb) [file 13023_2018_799_MOESM1_ESM.docx]

**Additional file 1. Detailed overview of CAPS symptoms and complications in 24 Dutch CAPS patients**

|  | Included in study (N=24) | Included in analyses (N=17) |
| --- | --- | --- |
| Symptoms |  |  |
| Fever | 13 (54.2%) | 9 (52.9%) |
| Skin rash | 20 (83.3%) | 15 (88.2%) |
| Musculoskeletal complaints | 21 (87.5%) | 15 (88.2%) |
| Arthralgia | 19 (79.2%) | 14 (82.4%) |
| Arthritis | 9 (37.5%) | 6 (35.3%) |
| Myalgia | 6 (25%) | 3 (17.6%) |
| Neck pain | 2 (8.3%) | 1 (5.9%) |
| Bursitis/Tendinitis | 2 (8.3%) | 2 (11.8%) |
| Back pain | 2 (8.3%) | 2 (11.8%) |
| Ocular symptoms | 13 (54.2%) | 10 (58.8%) |
| Conjunctivitis | 10 (41.7$) | 8 (47.1%) |
| Uveitis | 4 (16.7%) | 2 (11.8%) |
| Scleritis | 0 (0.0%) | 0 (0.0%) |
| Neurologic symptoms | 16 (66.7%) | 10 (58.8%) |
| Headache | 14 (58.3%) | 9 (52.9%) |
| Sterile meningitis | 3 (12.5%) | 2 (11.8%) |
| Papillitis | 1 (4.2%) | 0 (0.0%) |
| Papiledema | 1 (4.2%) | 1 (5.9%) |
| Collaps | 1 (4.2%) | 0 (0.0%) |
| Ulnaropathy | 1 (4.2%) | 1 (5.9%) |
| Polyneuropathy | 1 (4.2%) | 1 (5.9%) |
| Sensory neuropathy | 1 (4.2%) | 0 (0.0%) |
| Encephalomyelitis | 1 (4.2%) | 0 (0.0%) |
| Intercranial hypertension | 1 (4.2%) | 1 (5.9%) |
| Seizures | 1 (4.2%) | 1 (5.9%) |
| Scleritis | 0 (0.0%) | 0 (0.0%) |
| Mental retardation | 0 (0.0%) | 0 (0.0%) |
| Gastrointestinal symptoms | 8 (33.3%) | 5 (29.4%) |
| Abdominal pain | 4 (16.7%) | 2 (11.8%) |
| Diarrhoea | 4 (16.7%) | 2 (11.8%) |
| Vomiting | 2 (8.3%) | 1 (5.9%) |
| Nausea | 1 (4.2%) | 1 (5.9%) |
| Ructus | 1 (4.2%) | 1 (5.9%) |
| Epigastric pain | 1 (4.2%) | 1 (5.9%) |
| Lymphoreticular symptoms | 4 (16.7%) | 2 (11.8%) |
| Lymphadenopathy | 4 (16.7%) | 2 (11.8%) |
| Hepatomegaly | 0 (0.0%) | 0 (0.0%) |
| Splenomegaly | 0 (0.0%) | 0 (0.0%) |
| Cardiopulmonal symptoms | 3 (12.5%) | 1 (5.9%) |
| Chest pain | 2 (8.3%) | 0 (0.0%) |
| Dyspnea | 1 (4.2%) | 1 (5.9%) |
| Pleuritis | 0 (0.0%) | 0 (0.0%) |
| Pericarditis | 0 (0.0%) | 0 (0.0%) |
| Other symptoms | 8 (33.3%) | 5 (29.4%) |
| Aphtous ulcers | 3 (12.5%) | 2 (11.8%) |
| Loin pain | 2 (8.3%) | 2 (11.8%) |
| Fatigue | 2 (8.3%) | 1 (5.9%) |
| Swollen extremities | 1 (4.2%) | 0 (0.0%) |
| Pruritus | 1 (4.2%) | 0 (0.0%) |
| Rhinitis | 1 (4.2%) | 0 (0.0%) |
| Periocular edema | 1 (4.2%) | 1 (5.9%) |
| Retroperitoneal mass | 1 (4.2%) | 1 (5.9%) |
| Painful skin | 1 (4.2%) | 0 (0.0%) |
| Complications | 13 (54.2%) | 10 (58.8%) |
| Hearing loss | 9 (37.5%) | 7 (41.2%) |
| Decreased renal function/proteinuria | 5 (20.8%) | 3 (17.6%) |
| Hypertension | 4 (16.7%) | 3 (17.6%) |
| Cataract | 4 (16.7%) | 3 (17.6%) |
| Cardiovascular disease | 2 (8.3%) | 1 (5.9%) |
| Malignancy | 2 (8.3%) | 1 (5.9%) |
| Extrarenal AA amyloidosis | 2 (8.3%) | 2 (11.8%) |
| Persistent joint abnormalities | 1 (4.2%) | 0 (0.0%) |
| Glaucoma | 1 (4.2%) | 1 (5.9%) |
| Growth retardation | 1 (4.2%) | 1 (5.9%) |
